# Increased Excitability and Reduced Excitatory Synaptic Input Into Fast-Spiking CA2 Interneurons After Enzymatic Attenuation of Extracellular Matrix

**DOI:** 10.3389/fncel.2018.00149

**Published:** 2018-05-30

**Authors:** Hussam Hayani, Inseon Song, Alexander Dityatev

**Affiliations:** ^1^Molecular Neuroplasticity, German Center for Neurodegenerative Diseases (DZNE), Magdeburg, Germany; ^2^Center for Behavioral Brain Sciences (CBBS), Magdeburg, Germany; ^3^Medical Faculty, Otto-von-Guericke University, Magdeburg, Germany

**Keywords:** hippocampus, perineuronal net, WFA, fast-spiking interneuron, excitability, excitatory input

## Abstract

The neural extracellular matrix (ECM) is enriched with hyaluronic acid, chondroitin sulfate proteoglycans (CSPGs) and the glycoprotein tenascin-R, which play important roles in synaptic plasticity, as shown by studies of the CA1 region of the hippocampus. However, ECM molecules are strongly expressed in the CA2 region, which harbors a high number of fast-spiking interneurons (FSIs) surrounded by a particularly condensed form of ECM, perineuronal nets. Despite this intriguing peculiarity, the functional role of ECM in the CA2 region is mostly unknown. Here, we investigate the acute and delayed effects of chondroitinase ABC (ChABC), an enzyme that digests chondroitin sulfate side chains of CSPGs and greatly attenuates neural ECM, on neuronal excitability and excitatory transmission in the CA2 region. Whole-cell patch clamp recordings of CA2 pyramidal cells (PCs) and FSIs in hippocampal slices revealed that 7 days after injection of ChABC into the CA2 region *in vivo*, there are alterations in excitability of FSIs and PCs. FSIs generated action potentials with larger amplitudes and longer durations in response to less depolarizing currents compared to controls. PCs were excited at less depolarized membrane potentials, resulted in lower latency of spike generation. The frequency of excitatory postsynaptic currents in FSIs was selectively reduced, while the frequency of inhibitory postsynaptic currents was selectively increased. Acute treatment of hippocampal slices with ChABC did not result in any of these effects. This increase in excitability and changes in synaptic inputs to FSIs after attenuation of ECM suggests a crucial role for perineuronal nets associated with FSIs in regulation of synaptic and electrical properties of these cells.

## Introduction

The extracellular matrix (ECM) has a distinct role in brain development, maturation of neural circuits, and regulation of synaptic functions. Chondroitin sulfate proteoglycans (CSPGs), link proteins, hyaluronan and tenascin-R are well known components of ECM in the brain, which are particularly enriched in perineuronal nets (PNNs), covering soma, proximal dendrites and axon initial segments of parvalbumin (PV) expressing fast-spiking GABAergic interneurons and a subset of pyramidal cells (PCs) (Dityatev et al., 2007; Morikawa et al., 2017). Accumulating evidence suggests that the neural ECM supports structural stability and regulates synaptic plasticity, interacting with signaling molecules, membrane bound receptors and ion channels (Dityatev and Schachner, 2003; Frischknecht et al., 2009). PNNs are formed during the final stage of maturation of inhibitory neuronal circuits, which is particularly obvious in the rodent’s hippocampus during 2–3 weeks of postnatal development (Brückner et al., 2000). The elevated incorporation of CSPGs with a specific pattern of sulfation into PNNs may restrict CNS plasticity and lead to the end of the critical period in the postnatal CNS development, during which developmental forms of activity-dependent plasticity shape the functional circuitry (Carulli et al., 2007; Galtrey et al., 2008). The formation of PNNs at the end of the critical period coincides with the maturation of GABA interneurons and readjustment of the network excitatory/inhibitory (E/I) balance (Dityatev et al., 2010). Thus, it is of great interest to investigate if PNN manipulations could lead to alterations in E/I balance. Indeed, attenuation of ECM with the bacterial enzyme, chondroitinase ABC (ChABC), increased the excitability of fast-spiking interneurons (FSIs) selectively, without affecting PC’s intrinsic properties in the cultured hippocampal neurons (Dityatev et al., 2007). In this study, the reduced firing threshold and after-hyperpolarization were reported 1–2 days after ChABC treatment (Dityatev et al., 2007), which indicated for the first time that the PNNs may control excitability of FSIs.

Histochemical investigation of *Wisteria floribunda* agglutinin (WFA), brevican and aggrecan expression in rat and mouse brain reveals that CSPGs are highly enriched in the hippocampal CA2 area compared with CA1 and CA3 (Brückner et al., 2003; Ajmo et al., 2008; Mcrae et al., 2010; Carstens et al., 2016; Noguchi et al., 2017). Close examination of their expression patterns suggests that brevican is enriched in PNNs around PV-expressing (PV+) interneurons, while aggrecan protein and mRNA are detected in CA2 PCs and interneurons (Mcrae et al., 2010; Carstens et al., 2016). Recently, the CA2 region of the hippocampus received a lot of attention, and accumulating evidence suggests that it has its own unique cellular properties, function, and a distinctive role in learning and memory (Dudek et al., 2016). Interestingly, CA2 PCs and interneurons have unique molecular and cellular properties and network connectivity (Chevaleyre and Piskorowski, 2016). The CA2 area receives strong functional inputs from supramammillary nucleus, paraventricular nucleus and amygdala, as well as intrahippocampal inputs from dentate gyrus, CA3, and entorhinal cortex (Llorens-Martin et al., 2015; Chevaleyre and Piskorowski, 2016). CA2 PCs have a more hyperpolarized resting membrane potential and have a higher threshold for action potential generation when compared to CA1 and CA3 PCs due to the prominent expression of the two-pore-domain potassium channel, TREK-1 (Talley et al., 2001; Kohara et al., 2014; Piskorowski et al., 2016). It was recently shown that chronic silencing of CA2 PCs results in disruption of the E/I balance in the hippocampal circuits by weakening the CA2-mediated inhibition in CA3 (Boehringer et al., 2017). Moreover, genetic inactivation of CA2 PCs impairs social recognition memory (Hitti and Siegelbaum, 2014). CA2 PCs do not express conventional long-term potentiation (LTP), which can be easily observed in the CA1 area; however, these neurons are able to express vasopressin receptor/RGS14/A1 adenosine receptors-sensitive synaptic potentiation (Zhao et al., 2007; Lee et al., 2010; Simons et al., 2011; Chafai et al., 2012). Enzymatic attenuation of PNNs with ChABC in young mice allows re-establishment of high frequency stimulation-induced LTP in the CA2 area, implying that aggrecan-rich PNNs may restrict the plasticity of excitatory synapses on CA2 PCs (Carstens et al., 2016).

PV+ interneurons, as well as reelin-, calbindin- and calretinin-expressing interneurons, are more densely packed in the CA2 area than in other hippocampal areas (Piskorowski and Chevaleyre, 2013; Botcher et al., 2014). CA2 PV+ cells in rats show different dendritic morphologies, axonal arborizations, and electrophysiological properties (Mercer et al., 2012; Piskorowski and Chevaleyre, 2013) compared to PV+ neurons in other hippocampal subregions. The PV+ basket cells fire with a high frequency such as those in CA1; however, CA2 PV cells have horizontally oriented dendrites, adapted firing properties, and display prominent sag amplitude in response to hyperpolarizing voltage injection (Mercer et al., 2012). CA2 PV cells exhibit delta-opioid receptor-mediated LTD in area CA2, but not in CA1 (Piskorowski and Chevaleyre, 2013), despite delta-opioid receptors co-localization with PV+ cells uniformly through the whole hippocampus (Erbs et al., 2012).

Here, we investigated how attenuation of ECM differentially modulates the electrical properties, the excitatory and inhibitory transmission to two major types of CA2 neurons, PCs and FSIs. Acute treatment with ChABC did not affect neuronal excitability of these CA2 cells, but 7 days after *in vivo* injection of the enzyme, we observed an increase in FSI and PC excitability, and reduced excitatory and increased inhibitory transmission to FSIs. Our results highlight the significant role of ECM in regulation of synaptic and electrical properties of FSIs in hippocampal CA2 area *in vivo*.

## Materials and Methods

### Animals

Experiments were performed on young adult (3- to 5-week-old) C57Bl/6J mice bred at the SPF DZNE animal facility, all treatments were performed in accordance with ethical animal research standards regulated by Germany and were approved by the Ethical Committee on Animal Health and Care of the state of Saxony-Anhalt, Germany with a license number 42502-2-1322 and 42502-2-1159.

### Surgical Procedure and Stereotaxic Injection

Surgeries were performed, as previously described (Senkov et al., 2006; Kochlamazashvili et al., 2010). Briefly, mice were anesthetized with isoflurane (Baxter, Germany) and fixed in a stereotaxic instrument (SR-6M, Narishige Scientific Instrument Lab, Japan). The mice were placed on a heating pad connected to temperature controller (ATC1000, World Precision Instruments, USA) during the surgery process. The head was fixed, and the nose was held in an anesthetic mask for mice to provide oxygen and isoflurane using an isoflurane vaporizer (Matrx VIP 3000, Midmark, USA), followed by exposure of the skull. Two injections were done (one per hemisphere) at the following coordinates (in mm from bregma and the surface of the dura mater): Anterior-Posterior (AP) = +1.82; Medial-Lateral (ML) = ±1.95; Dorsal-Ventral (DV) = −1.5 with 400 nl of protease-free ChABC (50 U/ml; AMS.E 1028–10, AMSBIO, Europe) or control phosphate-buffered saline (PBS), as a vehicle, at 2 nl/s with 10 μl NanoFil syringe (World Precision Instruments, USA) using UltraMicroPump (UMP3, World Precision Instruments, USA). After injection, the needle was left for 5 min before being withdrawn. Mice were returned to the home cage and then observed until waking.

### Acute Hippocampal Slice Preparation

To study slow-developing (delayed) effects of ChABC, it was injected (50 U/ml) onto CA2 area *in vivo* 7 days before animals were decapitated. The brain was exposed, rapidly removed and chilled with ice-cold solution containing (in mM): 230 sucrose, 2.5 KCl, 7 MgCl_2_, 1.25 NaH_2_PO_4_, 26.2 NaHCO_3_, 0.5 CaCl_2_ and 10 D-Glucose (300 ± 5 mOsm). Coronal 350 μm-thick hippocampal slices were prepared with microtome (VT1200S, Leica, Germany) and were incubated in a submerged chamber at room temperature containing in mM: 113 NaCl, 2.38 KCl, 1.24 MgSO_4_, 0.95 NaH_2_PO_4_, 24.9 NaHCO_3_, 1 CaCl_2_, 1.6 MgCl_2_, 27.8 D-glucose and recovered for at least 1 h.

For acute ChABC treatment, acutely prepared hippocampal slices were incubated for 2 h with ChABC at 37°C, as described (Bukalo et al., 2001). Next, the slices were transferred to the recording chamber and were continuously perfused with a solution containing (in mM): 119 NaCl, 2.5 KCl, 1.3 MgSO_4_, 1 NaH_2_PO_4_, 26.2 NaHCO_3_, 2.5 CaCl_2_ and 11 D-Glucose (295 ± 5 mOsm). All solutions were saturated with 95% O_2_ and 5% CO_2_.

### Electrophysiology

Whole-cell recordings were made from CA2 PCs and FSIs visualized with an infrared differential interference contrast microscope (Slicescope, Scientifica, UK). Cell excitability was measured in the current-clamp mode while the membrane potential was clamped at −70 mV using a glass pipette (3–5 MΩ, Hilgenberg, Germany) containing (in mM): 140 K-gluconate, 8 NaCl, 0.2 CaCl_2_, 10 HEPES, 2 EGTA, 0.5 NaGTP and 2 MgATP (pH 7.2 with KOH, 290 mOsm). To provide examples of morphology of recorded neurons, the glass pipettes were filled with intracellular recording solution containing 0.5% biocytin (Sigma-Aldrich, Germany). The serial resistance was routinely monitored and only cells with serial resistance less than 20 MΩ and leak current <100 pA and >−100 pA were included in analysis. Current injections were performed with the duration of 500 ms and increasing in amplitude from −80 pA to 520 pA with a 40 pA current step to elicit action potentials and estimate the firing threshold. For measuring electrophysiological properties of action potentials, sweeps were used in which first action potential(s) appear. The amplitude of action potential was calculated as the peak amplitude above the membrane potential at which an action potential was generated. The after-hyperpolarization was measured as the difference between the membrane potential at which the action potential was generated and the local minimum following the action potential. The half width of action potential was computed as the time interval between the action potential rising to 50% and falling to 50% of its peak amplitude. The rise slope was calculated within the time taken for the action potential to rise from 20% to 80% of its peak amplitude, whereas the decay slope was calculated within the time taken for the action potential to decay from 80% to 20% of its peak amplitude. The firing threshold was calculated as the minimum depolarizing current required to elicit the first action potential. The membrane potential threshold was determined as the membrane potential value at which the change of the membrane potential (d*V*/d*t*) exeeded 20 mV/ms. Action potential adaptation was measured as a ratio of the intervals between the first two and following pairs of action potentials generated at 480 pA and 520 pA current step for FSIs and PCs, respectively. The latency of action potential was considered as the time taken from the onset of stimulus to reach the peak amplitude of the first action potential. First voltage steps, where action potentials have not appeared yet, were used to measure the input resistance. After recording of action potentials, spontaneous sEPSCs were acquired in the voltage-clamp mode, with the membrane potential clamped at −70 mV i.e., at resting membrane potential. The amplitude of sEPSC was measured as the difference between the baseline and the peak of sEPSC. The sEPSC 10%–90% rise time was considered as the time taken for the sEPSC to rise from 10% to 90% of its peak amplitude; whereas the sEPSC decay time was considered as the time taken for the sEPSC to decay from 90% to 10% of its peak amplitude. The frequency of sEPSCs was calculated by counting the frequency of sEPSCs occurrence within 5 min. Miniature EPSC (mEPSC) measurements were done at −70 mV, with a glass electrode containing (in mM): 130 K-gluconate, 8 NaCl, 10 HEPES, 2 EGTA, 5.8 QX314-Cl, 3 L-ascorbic acid, 0.5 Na_2_GTP and 2 MgATP (pH 7.2 with KOH, 290 mOsm) in the presence of tetrodotoxin (TTX, 1 μM), picrotoxin (50 μM), and CGP 55845 (2 μM). For sIPSCs recordings at −70 mV, the intracellular solution contained (in mM): 120 Cesium Methanesulfonate, 8 NaCl, 0.2 MgCl_2_, 10 HEPES, 2 EGTA, 0.3 Na_2_GTP and 2 MgATP. NBQX (20 μM), D-AP5 (50 μM) and CGP 55845 (2 μM) were added to bath. TTX was additionally dissolved to bath for mIPSC recording. Properties of mEPSC, sIPSC, and mIPSC were analyzed with MiniAnalysis (Synaptosoft Inc., USA).

Potassium (K^+^) currents were measured in the presence of TTX using glass pipette containing (in mM) 140 KCl, 10 EGTA, 2 MgCl_2_, 2 Na_2_ATP, and 10 HEPES (pH 7.3 with KOH, 290 mOsm). Composite K^+^ currents were measured in the voltage-clamp configuration by delivering voltage steps ranging from −40 mV to +40 mV with 10 mV increment, following a potential step of 200 ms to −100 mV. All experiments were performed at 30–31°C. Tetrodoxin, picrotoxin, CGP55845, D-AP5 and NBQX were purchased from Tocris.

### Immunohistochemistry

All hippocampal slices used for the electrophysiological recording were subjected to WFA labeling to assure that ChABC properly attenuated the ECM of PNNs. The 350 μm slices were fixed in 4% paraformaldehyde (PFA) overnight. The slices were washed three times with PBS, cryoprotected with 30% sucrose in PBS, freeze-thawed, rinsed three times in 0.25% goat serum in PBS for 20 min each, and incubated in PBS containing 5% goat serum and 0.2% Triton X-100 for 1 h to block nonspecific binding. Slices were incubated overnight at 4°C in biotinylated WFA (L1516, Sigma, 1:400) in PBS containing 2.5% normal goat serum. The day following incubation, slices were reacted with streptavidin-conjugated Alexa 488 (S11223, Invitrogen, 1:300) for 1 h at room temperature. The slices were washed three times with PBS for 10 min each before being mounted in mounting medium (Vectashield H-1000, Vector laboratories Inc., USA) on glass slides. Slides were examined with an upright laser scanning confocal microscope (LSM 700, Carl Zeiss, USA). The same preparation was used for *post hoc* immunohistochemical identification of recorded biocytin-labeled neurons, with the addition of the following primary antibodies: mouse anti-RGS14 (ab118353, Abcam, 1:100) and rabbit anti-PV (PV27, SWANT Swiss Antibodies, 1:300). The day following incubation, slices were incubated with Alexa Fluor 546 goat anti-mouse and Alexa Fluor 647 goat anti-rabbit antibodies (both from Invitrogen, 1:300). For quantitative immunohistochemical analysis of perisomatic synaptic contacts onto PV+ interneurons in CA2 region 7 days after injection with either ChABC (*n* = 3) or PBS (*n* = 3), mice were perfused transcardially with 40–45 ml ice-cold PBS followed with 40–45 ml ice-cold 4% PFA. Then, brains were extracted and post-fixed overnight in 4% PFA at 4°C and subsequently cryoprotected with 30% sucrose in PBS for two nights at 4°C. Until the day of cryostat sectioning, brains were frozen at −80°C. The brains were cut using a cryostat (Leica CM 1950, Leica Biosystems Nussloch GmbH, Germany) to obtain 50 μm coronal sections. Free floating sections were rinsed in PBS and then permeabilized in 0.5% Triton X-100 in PBS for 30 min followed with blocking solution (10% normal goat serum, 0.1% glycine and 0.4% Triton X-100 in PBS) for 6 h at RT. Sections were then incubated with primary guinea pig anti-VGluT1 (135304, Synaptic systems, 1:500) and chicken anti-PV antibodies (195006, Synaptic systems, 1:500) for 40 h at 4°C. After washing with PBS three times, sections were incubated with secondary antibodies: Alexa Fluor 488 goat anti-guinea pig (A11073, 1:1000, Invitrogen) and Alexa Fluor 647 donkey anti-chicken (AP194SA6, Millipore Sigma, 1:1000) for 3 h at RT. After a wash with PBS, sections were placed on glass slides and left to dry for 10 min at RT before being mounted with fluoromount (Sigma-Aldrich, Germany).

### Data Acquisition and Analysis

Recordings were obtained using an EPC-10 amplifier (Heka Electronik, Lambrecht/Pfalz Germany). The recordings were filtered at 2–5 kHz and digitized at 10–20 kHz. Electrophysiological data were analyzed with MiniAnalysis (Synaptosoft Inc., USA) and Clampfit 10.0 (Molecular device, USA). Statistical analysis was performed using Sigmaplot 12.3 (Systat software Inc., USA). All data are presented as the mean ± SEM, and the significant difference between groups was estimated with Mann-Whitney U-test, if the assumption of normality of distributions failed, or unpaired *t*-test, as stated in the text. The *P*-values smaller than 0.05 were considered as statistically significant.

For quantitative immunohistochemical analysis of VGluT1 puncta, laser scanning confocal microscope (LSM 700, Carl Zeiss) was used to acquire images with 63× oil immersion objective. Confocal z-stacks covering the first 3 μm in z-axis were taken at acquisition intervals of 0.3 μm. A single plane from each section (1.5–2.1 μm from the slice surface) was used to quantify VGluT1 perisomatic puncta onto PV positive interneurons in the CA2 region using Fiji software. Somatic profiles of these interneurons were outlined and only puncta located within a distal distance of 0.6 μm of the somatic profile were counted. The number of puncta and perimeter from each interneuron were determined and mean values for each animal were used for statistical comparison. The density of puncta was computed as the number of puncta per 10 μm of soma perimeter.

## Results

### ECM Digestion by ChABC Injection in the CA2 Region of the Hippocampus

**Figure 1:**
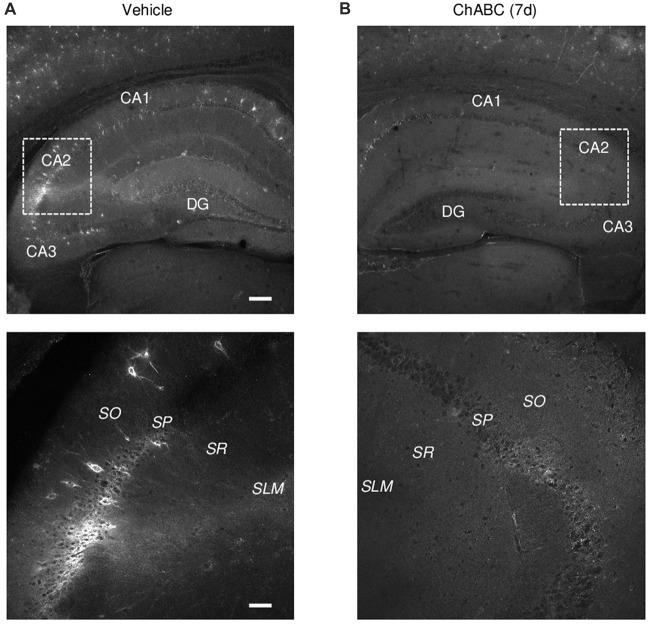
*Wisteria floribunda* agglutinin (WFA) labeling of perineuronal nets (PNNs) 7 days (7 d) after intrahippocampal chondroitinase ABC (ChABC) injection. **(A)** WFA staining in a vehicle-injected hippocampus shows a strong labeling of PNNs with enrichment in the CA2 region. **(B)** WFA staining was abolished 7 days after ChABC injection in CA2. Scale bars, 200 and 50 μm in the upper and lower rows, respectively.

To investigate the role of ECM in the CA2 region, we either applied ChABC to acute hippocampal slices for 2 h (Bukalo et al., 2001) or bilaterally injected ChABC into the CA2 region *in vivo* and performed recordings in 7 days *ex vivo* (Yang et al., 2015). To visualize a loss of ECM after ChABC treatment, we used WFA labeling of PNNs (Brauer et al., 1993; Brückner et al., 1994; Seeger et al., 1996). Noteworthy, WFA staining in control slices revealed the high level of PNNs expression in the CA2 region compared to PNNs expression in CA1 and CA3 regions (Figures 1A,B). As demonstrated previously (Bukalo et al., 2001), 2 h treatment of slices with the chosen concentration of ChABC fully ablated WFA labeling in hippocampal slices (data not shown). Seven days after ChABC injection in the CA2 region, PNNs were strongly attenuated, not only in CA2 but also in other hippocampal areas, compared to vehicle injected hippocampal slices (Figures 1A,B). This is not surprising considering known diffusion properties and stability of the enzyme at physiological conditions. Thus, both *in vitro* and *in vivo* treatment with ChABC removed WFA-positive ECM in the region of interest.

### The Effects of ChABC Treatment on the Excitability of CA2 Pyramidal Cells

**Figure 2:**
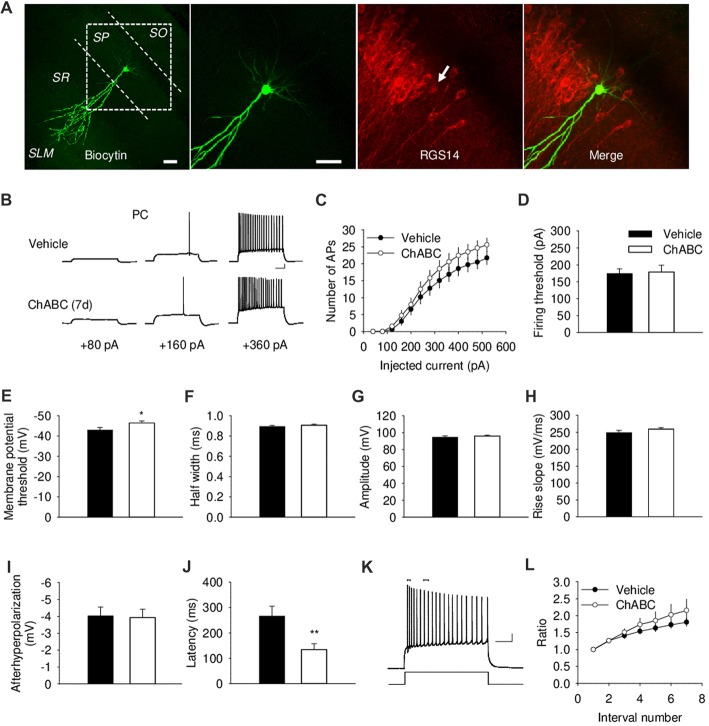
Basic electrical properties of CA2 pyramidal cells (PCs) 7 days (7 d) after intrahippocampal injection of ChABC. **(A)** Intracellular labeling of the recorded CA2 PC. Scale bar, 50 μm. **(B)** Sample traces of action potentials (APs) recorded in the CA2 PC in response to depolarizing current injections 7 d after vehicle/ChABC injection. Scale bar, 10 mV/100 ms. **(C)** Summary graph showing the number of action potentials (APs) elicited in CA2 PCs in response to step-wise depolarizing currents of increasing amplitude. Two-way RM ANOVA, ChABC effects: *P* > 0.05. Bar graphs comparing ChABC- (*n* = 20) vs. vehicle-treated (*n* = 18) CA2 PCs in terms of the firing threshold **(D)**, the threshold depolarization necessary for action potential generation **(E)**, the half-width of action potentials **(F)**, the amplitude of action potentials **(G)**, the rise slope of action potentials **(H)**, the after-hyperpolarization of action potentials **(I)**, the action potential latency of PCs **(J)**. **(K)** An example of action potential adaptation. Scale bars, 10 mV/100 ms. Upper bars indicating the first and last intervals used to calculate action potential adaptation. **(L)** Action potential adaptation, measured as a ratio of the interval between the first two and following pairs of action potentials generated at 520 pA current step. Bars represent mean ± SEM values. *P < 0.05, **P < 0.01, *t*-test, compared with vehicle.

To investigate whether the acute and/or slowly developing effects of ECM deficiency on electrical properties of CA2 PCs, we carried out whole-cell current-clamp recordings. We identified the CA2 region visually as the region between CA1 and CA3 regions with thick cell body layer (Chevaleyre and Siegelbaum, 2010). The proper location and identity of the recorded cells was confirmed by adding biocytin to the intracellular solution for *post hoc* cell visualization, in combination with CA2-specific marker RGS14 (Figure 2A and Supplementary Figure S1A). CA2 PCs, unlike those in CA1, have large somata and distinct dendritic morphology with the apical dendrite of CA2 neurons bifurcating close to the cell body and extending to the *stratum lacunosum-moleculare*, where they further branch (Ishizuka et al., 1995; Mercer et al., 2007; Chevaleyre and Siegelbaum, 2010; Figure 2A and Supplementary Figure S1A). CA2 PCs have distinct electrophysiological characteristics manifested in action potentials with a half-width of approximately 1 ms and prominent adaptation of action potential frequency (Figure 2B). Our electrophysiological analysis of action potentials under ChABC treatment revealed no acute or delayed effects on the number of action potentials generated by multiple steps of depolarizing current, firing threshold, half-width, amplitude, rise slope, after-hyperpolarization of action potentials or spiking adaptation, when compared to cells from control slices (Figures 2B–L and Supplementary Figures S1B–S1D). The input resistance was also not affected (data not shown). The only two differences that we observed between groups 7 days after enzyme injection were a slight alteration in the minimum level of membrane depolarization necessary to elicit an action potential (Vehicle: −43 ± 1.18 mV; ChABC: −46.4 ± 1.04 mV, *P* < 0.05) and a corresponding decrease in action potential latency (Vehicle: 266.1 ± 38.7 ms; ChABC: 134.5 ± 102.5 ms, *P* < 0.01).

### Delayed Effects of ChABC Treatment on Electrical Properties of CA2 Fast-Spiking Interneurons

**Figure 3:**
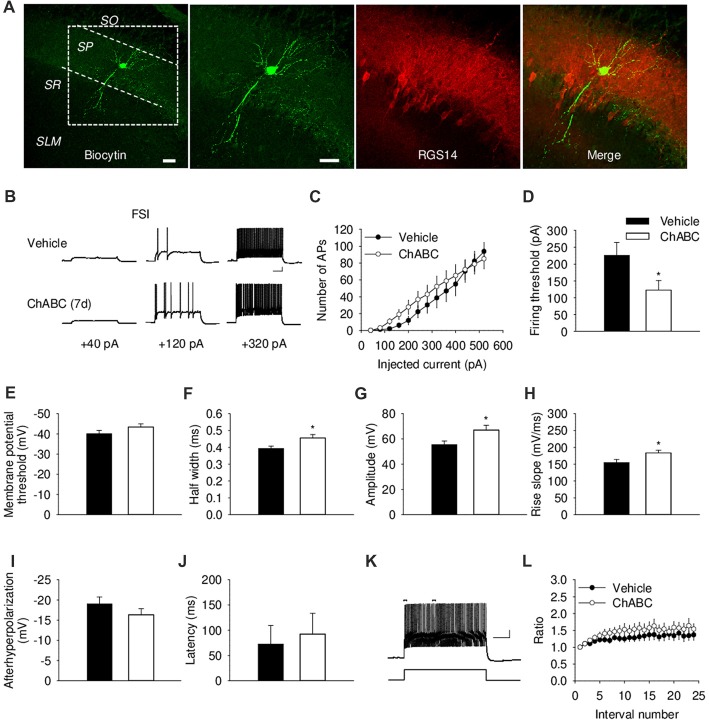
Basic electrical properties of CA2 fast-spiking interneurons (FSIs) 7 days (7 d) after intrahippocampal injection of ChABC.** (A)** Intracellular staining of the recorded CA2 FSI. Scale bar, 50 μm. **(B)** Sample traces of action potentials in the CA2 FSI. Scale bars, 10 mV/100 ms. **(C)** Number of action potentials elicited in CA2 FSIs in response to multiple steps of depolarizing current. Two-way RM ANOVA, effects of ChABC: *P* > 0.05. Bar graphs comparing ChABC- (*n* = 11) vs. vehicle-treated (*n* = 12) FSIs in terms of the firing threshold **(D)**, the threshold depolarization necessary for action potential generation **(E)**, the half-width of action potentials **(F)**, the amplitude of action potentials **(G)**, the rise slope of action potentials **(H)**, the afterhyperpolarization of action potentials **(I)**, the action potential latency of PCs **(J)**. Bars represent mean ± SEM. *P < 0.05, unpaired *t*-test/U-test, compared with vehicle. **(K)** An example of action potential adaptation. Scale bars, 10 mV/100 ms. Upper bars indicating the first and last intervals used to calculate action potential adaptation. **(L)** Action potential adaptation, measured as a ratio of the intervals between the first two and pairs of following action potentials generated at 480 pA current step.

We further investigated the effect of ECM digestion by ChABC treatment on the electrical properties of CA2 FSIs, which were electrophysiologically identified by having action potentials with the half-width <0.5 ms and spiking frequency >40 Hz and that showed minimal spiking adaptation (Figure 3; Dityatev et al., 2007; Mercer et al., 2007, 2012). Intracellular pipette solution contained biocytin to allow visualization of the recorded FSIs (Figure 3A and Supplementary Figure S1A), which exhibited morphological characteristics of CA2 basket cells with a characteristic pattern of dendritic arborizations and axonal swellings (putative synaptic boutons) in the pyramidal cell layer. The patch-clamp recording of action potentials from CA2 FSIs revealed alterations in several electrophysiological parameters 7 days after ChABC injection. The FSIs evoked action potentials in response to the ≈50% less depolarizing currents compared to the control, indicating a lower firing threshold upon PNNs removal (Figure 3D). Additionally, the action potentials had ≈16% longer half-width (Figure 3F) and ≈21% larger amplitude (Figure 3G), as well as ≈18% greater rise slope (Figure 3H); whereas other parameters, such as after-hyperpolarization amplitude (Figure 3I), latency of action potential (Figure 3J), action potential frequency adaptation (Figures 3K,L) and cell input resistance (data not shown) were not altered compared to the control. No difference was detected in either the amplitude or the activation kinetics of K^+^ currents (Supplementary Figure S2). On the other hand, our electrophysiological analysis of FSIs after acute ChABC treatment detected no difference in any measured parameter compared to vehicle-treated controls (Supplementary Figures S1E–S1G).

### Delayed Effects of ChABC Treatment on Excitatory Synaptic Transmission to CA2 Fast-Spiking Interneurons

**Figure 4:**
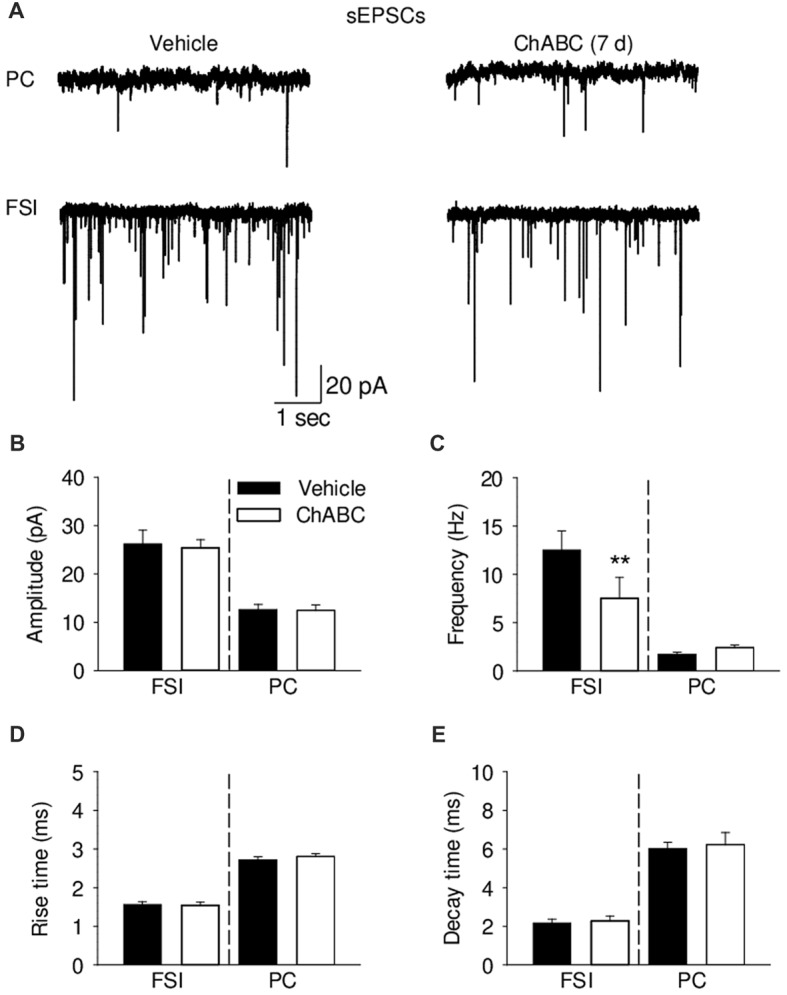
Analysis of excitatory synaptic transmission onto pyramidal cells and fast-spiking interneurons 7 days (7 d) after intrahippocampal injection of ChABC. **(A)** Representative traces of spontaneous excitatory post-synaptic currents (sEPSCs) from CA2 PCs (upper trace) and FSIs (lower trace) in vehicle- and ChABC-injected mice. Bar graphs comparing ChABC- vs. vehicle-treated CA2 FSIs and PCs in terms of the sEPSC amplitude **(B)**, frequency **(C)**, rise time **(D)** and decay time **(E)**. Bars represent mean ± SEM values in vehicle- and ChABC-treated FSIs (Vehicle, *n* = 11; ChABC, *n* = 11) and PCs (Vehicle, *n* = 11; ChABC, *n* = 11), **P < 0.01, U-test, compared with vehicle.

**Figure 5:**
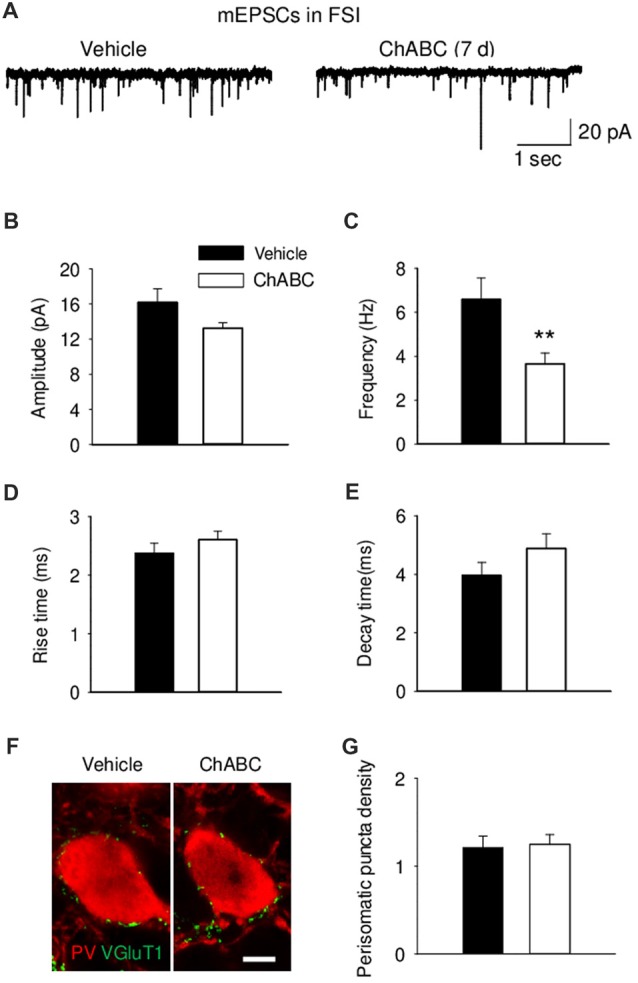
Decreased frequency of action-potential independent miniature EPSCs (mEPSCs) onto fast-spiking interneurons 7 days (7 d) after ChABC injection. Representative traces **(A)**, amplitude **(B)**, frequency **(C)**, rise **(D)** and decay time **(E)** of mEPSCs in FSIs after Vehicle (*n* = 9) or ChABC (*n* = 9) treatments. **(F)** Graph representing VGluT1 expression in the perisomatic region of parvalbumin (PV)-expressing FSIs. Scale bar, 5 μm. **(G)** Perisomatic puncta density (number of puncta/10 μm of soma perimeter) after treatment with Vehicle (33 neurons from three mice) or ChABC (39 neurons from three mice). Bars represent mean ± SEM values (per animal) in vehicle- (black) and ChABC-treated (white) groups. **P < 0.01, *t*-test, compared with vehicle.

To study whether digestion of ECM could alter glutamatergic transmission to CA2 neurons, we recorded the sEPSCs from CA2 PCs and FSIs after ChABC treatment (Figure 4 and Supplementary Figure S3). The typical characteristics of sEPSCs differed between PCs and FSIs, as previously reported (Povysheva et al., 2006). PCs exhibited sEPSCs with smaller amplitude, slower rise-time and decay-time, and lower frequency than sEPSCs recorded in FSIs (Figure 4 and Supplementary Figure S3). Acute ChABC treatment affected sEPSCs neither in PCs nor in FSIs (Supplementary Figure S3). However, there was a delayed effect of ChABC treatment on sEPSCs recorded in FSIs 7 days after injection; the frequency was reduced by ≈40% compared to control (Figure 4C). The amplitude, rise time, and decay time of sEPSCs remained intact (Figures 4B,D,E). Next, we pharmacologically isolated action potential-independent mEPSCs (Figures 5A–E and Supplementary Figure S4). A similar delayed effect of ChABC treatment was observed on the frequency of mEPSCs in FSIs, as was found for sEPSCs (Figure 5C). The amplitude, rise time, and decay time of mEPSCs remained intact (Figures 5B,D,E). The acute ChABC treatment had no effect on mEPSCs in both PCs and FSIs (Supplementary Figures S4B–S4E). Next, we investigated whether this delayed effect on excitatory transmission may be attributed to an alteration of excitatory innervation of FSIs. Thus, we carried out quantitative immunohistochemical analysis of perisomatic excitatory synaptic contacts onto FSIs 7 days after ChABC injection by double immunostaining of PV as FSIs marker and VGluT1 as excitatory presynaptic marker (Figures 5F,G). Density of perisomatic puncta remained intact, which indicates no loss of excitatory inputs onto FSIs after ChABC treatment.

### Delayed Effects of ChABC Treatment on Inhibitory Synaptic Transmission in the CA2 Region

**Figure 6:**
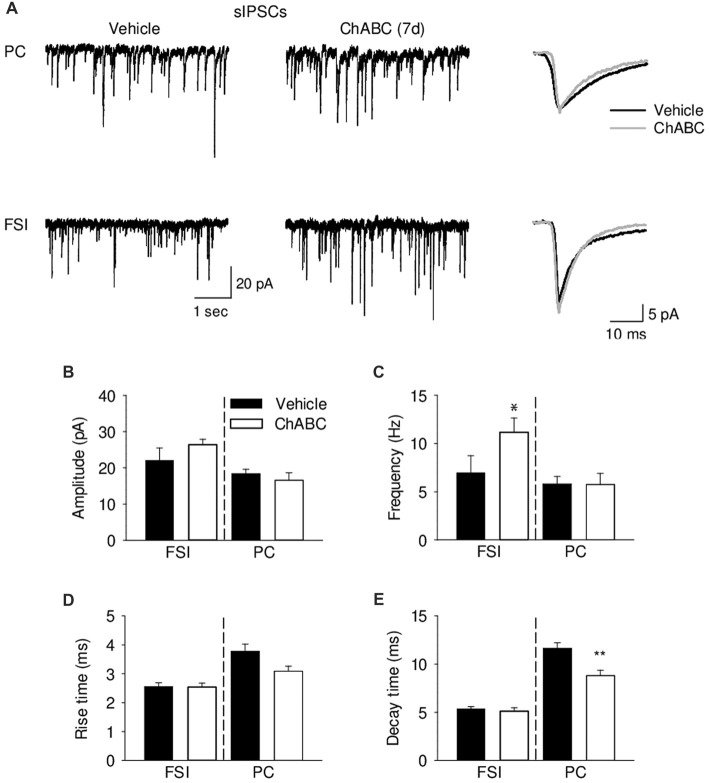
Analysis of GABAergic transmission onto pyramidal neurons and fast-spiking interneurons 7 days (7 d) after ChABC injection. **(A)** Representative traces of sEPSC from CA2 pyramidal cells (upper) and FSIs (lower) in vehicle- and ChABC-injected hippocampal slices. Bar graphs of sIPSC amplitude **(B)**, frequency **(C)**, rise time **(D)** and decay time **(E)** of CA2 PCs and FSIs. Bars represent mean ± SEM in vehicle- (black) and ChABC-treated (white) FSIs (Vehicle, *n* = 9; ChABC, *n* = 14) and PCs (Vehicle, *n* = 14; ChABC, *n* = 10). *P < 0.05, *t*-test after log-transformation to normalize distributions; **P < 0.01, *t*-test, compared with vehicle.

To verify whether ECM attenuation may modulate inhibitory inputs to CA2 neurons, we recorded pharmacologically isolated sIPSCs from CA2 PCs and FSIs 7 days after ChABC treatment (Figure 6). Consistently with the lowered firing threshold of FSIs and a known high degree of inhibitory coupling between basket cells, there was ≈40% increase in the sIPSC frequency after ChABC treatment as compared to control. In PCs, we detected no increase in the sIPSC frequency (Figure 6C) but observed ≈25% faster decay time of sIPSCs (Figure 6E). The amplitude and rise time of sIPSCs remained unchanged (Figures 6B,D). Acute ChABC treatment did not affect mIPSCs recorded in PCs and in FSIs (Supplementary Figure S5).

## Discussion

Our data revealed specific alterations in the excitability and synaptic inputs to CA2 FSIs and PCs 7 days after ChABC treatment, while no acute effects of ChABC were detected. An obvious importance of these findings is in the focus on the CA2 neurons, as previous studies mostly investigated the effects of enzymatic treatments and genetic loss of ECM molecules in the CA1, CA3 and dentate gyrus (Bukalo et al., 2001; Brakebusch et al., 2002; Evers et al., 2002; Gurevicius et al., 2009; Favuzzi et al., 2017).

Our results imply that removal of ECM with ChABC reduces the current threshold necessary for action potential generation in FSIs, but not in PCs. This is consistent with the previous analysis of cultured hippocampal neurons (Dityatev et al., 2007), although the effects observed in the present study are more prominent and also additional parameters of action potentials are affected, which may be related to the differences in timing and the experimental systems used (7 days after *in vivo* treatment vs. 1–2 days *in vitro* treatment). In CA2 PCs, the voltage threshold of spike generation was altered, which facilitated earlier generation of the first spike upon cell depolarization. The observed changes in excitability could be related to the observation that WFA-positive PNNs in the rodent hippocampus are mostly located around the soma of FSIs. However, in the CA2 region, ECM is highly enriched, and PNNs are also observed around PCs (Nakagawa et al., 1986; Celio, 1993; John et al., 2006; Carstens et al., 2016). It suggests that the observed, cell type-specific alterations in excitability are due to some differences between PCs and FSIs in ECM cell surface receptors and/or related intracellular signaling, rather than ECM enrichment *per se*.

Which ECM molecules are responsible for the modulation of FSIs excitability? CSPGs, brevican, neurocan and aggrecan are highly expressed within the hippocampus (Brückner et al., 2003). Genetic deletion of brevican and neurocan in the mice impair Schaffer collateral stimulated CA1 LTP in the hippocampal slices, implying that these proteoglycans are important for maintaining hippocampal synaptic plasticity (Zhou et al., 2001; Brakebusch et al., 2002). A very recent study demonstrated that dynamic change of the excitatory transmission and firing properties of PV+ cells depend on brevican expression in the hippocampal CA1 region (Favuzzi et al., 2017). We assume that a similar brevican-dependent mechanism may operate in the CA2 region.

Alterations in excitability of the neurons could be due to the regulation of voltage- or ligand-gated ion channel expression, their density, position and composition (Grubb and Burrone, 2010; Kuba et al., 2010; Davis, 2013). Currently, there are several ECM molecules that are already known to be involved in the regulation of some ion channels, such as tenascin-C and tenascin-R, which regulate sodium channels (Srinivasan et al., 1998). Moreover, tenascin-C, hyaluronic acid and integrins regulate L-type calcium channels and potassium channels (Davis et al., 2002; Evers et al., 2002; Kochlamazashvili et al., 2010). Furthermore, the axon initial segment is associated with PNNs molecules, especially brevican (John et al., 2006), and has a crucial role in neuronal excitability depending on its position and composition. A recent study suggests that heparan sulfate proteoglycans regulate integrity of the distal part of the AIS (Minge et al., 2017). When the AIS is closer to the soma, the firing threshold needed to evoke action potentials is lower (Grubb and Burrone, 2010). Several types of voltage-gated ion channels are highly enriched in the AIS, including potassium channels K_v_3. Therefore, manipulation of PNNs could shift the AIS distance from the soma and/or alter the composition of voltage-gated ion channels in the AIS, leading to an alteration in the neuronal excitability and synaptic properties. It is also plausible that changes in outflow of K^+^ from postsynaptic cells may induce modification in cell excitability (Dityatev and Schachner, 2003), as PNNs has highly polyanionic nature and, thus, could form a cation buffering system for cations, such as Ca^2+^ and K^+^ (Brückner et al., 1993; Hartig et al., 1999). The high spiking activity of FSIs could be attributed to cation channels, particularly the voltage-gated delayed rectifier channels subunit Kv3.1b (Karetko and Skangiel-Kramska, 2009), which would affect the firing properties of these interneurons (Dityatev et al., 2007). However, the total K^+^ current in our study was not altered after attenuation of PNNs by ChABC injection (Supplementary Figure S2), suggesting that specific regulation of axonal K^+^ channels may be involved. This is consistent with a recent report showing that brevican regulates cellular and synaptic plasticity in PV+ cells by modulating the localization of Kv3.1 potassium channels in the CA1 (Favuzzi et al., 2017).

Chondroitin sulfate-rich ECM binds to diverse proteins and may regulate cellular functions via a multitude of mechanisms. For instance, the alteration in neuronal excitability could be attributed to a shift in E/I balance, driven by the homeoprotein orthodenticle homeobox protein 2 (Otx2), which internalization into FSIs depends on PNNs. A recent study revealed a key role of Otx2 in maturation of PV+ GABA interneurons and the closure of the critical period of developmental plasticity in the visual cortex (Courtois et al., 2003; Beurdeley et al., 2012; Bernard and Prochiantz, 2016). Thus, PNN-assisted Otx2 internalization process may increase the perisomatic inhibition of PCs, leading to a shift in the E/I balance (Prochiantz, 2013). Although there are no data on the functional role of Otx2 specifically within the hippocampus, it is plausible to assume on basis of cortical data that attenuation of PNNs by ChABC, which reduces Otx2 uptake by FSIs and, thus, reopens the critical period of these neurons and changes the FSIs excitability and excitatory innervation.

CSPGs, the main target of ChABC, interfere with integrin signaling (Wu et al., 2002; Tan et al., 2011). ChABC treatment enhances β1-integrin activation that in turn triggers the phosphorylation of focal adhesion kinase (FAK), its downstream effector. This activation/phosphorylation process enhances dendritic spine motility and spine head protrusion formation (Stanco et al., 2009; Orlando et al., 2012). Thus, enzymatic removal of CSPGs may influence the functional properties of FSIs through integrin signaling.

The neuronal surface receptors, such as receptor protein tyrosine phosphatase σ (RPTPσ) and its homolog leukocyte common antigen-related phosphatase (LAR), which serve as receptors for regeneration-inhibiting glycosylated side chains of CSPGs (Shen et al., 2009; Fisher et al., 2011), may also be involved in the mechanism by which CSPGs removal influences the neuronal electrical properties and/or synaptic function. Indeed, a recent study in rats, in which treatment with ChABC cleaved the glycosylated CSPG side chains, relieved CSPG-mediated inhibition through RPTPσ and allowed axonal regeneration (Lang et al., 2015) through a mechanism in which heparan sulfates, competitor binders to RPTPσ that have an opposite effect to chondroitin sulfates, allowed RPTPσ clustering and inactivation (Coles et al., 2011).

In parallel with the enhanced excitability, we observed the reduced frequency of sEPSCs as well as mEPSCs in FSIs. Because the density of VGluT1 immunopositive presynapses onto FSI somata was not altered, we think that reduction in frequency of sEPSCs/mEPSCs results from impaired pre- or most likely postsynaptic function. Indeed, a previous *in vitro* study revealed a loss of neuronal pentraxins and GluA4 subunit expression after ChABC treatment (Chang et al., 2010). A similar reduction in EPSC frequency, as found in the present study, was detected in brevican deficient mice in the CA1 region (Favuzzi et al., 2017), which correlated with decreased expression of GluA1 in synaptosomes and lower number of synaptic inputs. In contrast, acute *in vitro* ChABC treatment of hippocampal slices for 2 h did not significantly alter neuronal firing properties and excitatory synaptic transmission of CA2 neurons, although frequencies of both sEPSCs and mEPSCs were reduced by 30%. Thus, it is plausible to assume that slow homeostatic mechanisms may arise to oppose the reduction of excitatory input to FSIs by elevation of their excitability upon treatment with ChABC (Dityatev et al., 2010).

Our analysis of inhibitory inputs to PCs and FSIs revealed an increase in frequency of sIPSCs in FSIs and faster sIPSC decay time in PCs. The former may reflect facilitated generation of spikes by FSIs after ChABC treatment. The latter may be attributed to decreased GABA level in the extracellular space upon ECM attenuation, decreased GABA release by inhibitory interneurons or changes in composition of GABA_A_ receptors.

In summary, our findings of increased excitability and inhibitory interactions between FSIs, in combination with reduction of excitatory input to FSIs after ECM attenuation in the CA2 region highlight a pivotal role for PNNs in regulation of synaptic and electrical properties of FSIs. Strikingly, also excitability of PCs, many of which are surrounded by PNNs in CA2, was modulated 7 days after ChABC injection. In summary, our findings revealed that attenuation of ECM induces complex reorganization of PC-FSI neuronal network in the CA2 region. More studies are warrantied to uncover the underlying cellular and molecular mechanisms.

## Author Contributions

AD and IS designed and supervised the research. HH and IS performed this research, analyzed data and wrote the first draft. All authors edited the manuscript.

## Conflict of Interest Statement

The authors declare that the research was conducted in the absence of any commercial or financial relationships that could be construed as a potential conflict of interest.
