# Supplementary material for: Increased Excitability and Reduced Excitatory Synaptic Input Into Fast-Spiking CA2 Interneurons After Enzymatic Attenuation of Extracellular Matrix
Source: Front Cell Neurosci. 2018 May 30;12:149. doi: 10.3389/fncel.2018.00149 (PMC5988902; doi:10.3389/fncel.2018.00149)
Supplement: Supplementary file 1 [file Presentation_1.pdf]

Supplementary material

Increased excitability and reduced excitatory synaptic input into fast-spiking CA2 interneurons after enzymatic attenuation of extracellular matrix

Hussam Hayani[1,+], Inseon Song[1,+], Alexander Dityatev[1,2,3*]

[1]Molecular Neuroplasticity, German Center for Neurodegenerative Diseases (DZNE), 39120 Magdeburg, Germany

[2]Center for Behavioral Brain Sciences (CBBS), 39120 Magdeburg, Germany

[3]Medical Faculty, Otto-von-Guericke University, 39120 Magdeburg, Germany

[+]Equally contributing authors

*To whom correspondence should be addressed:

Prof. Alexander Dityatev, PhD

German Center for Neurodegenerative Diseases (DZNE)
Leipziger Str. 44, Haus 64
39120 Magdeburg
Germany

Tel.: +49 391 67 24526

Fax: +49 391 6724530

E-mail: Alexander.Dityatev@dzne.de

Short title: ECM functions in CA2

Keywords: hippocampus, perineuronal net, WFA, fast-spiking interneuron, excitability, excitatory input

Supplementary figures

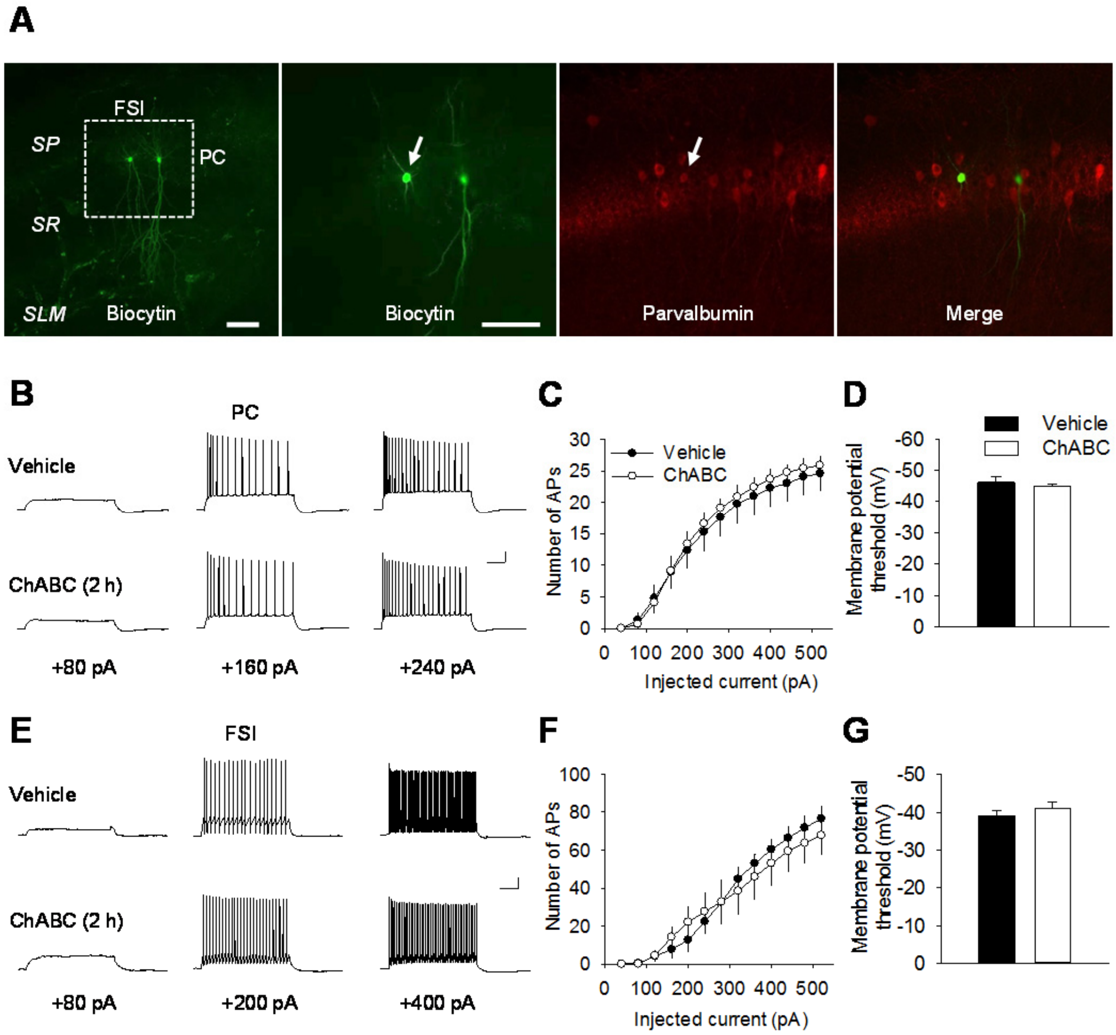

Supplemental Figure S1. Acute ChABC treatment does not affect excitability of CA2 neurons. **A**. Intracellular labeling of recorded CA2 PC and FSI and parvalbumin immunostaining. An arrow points to a recorded cell with electrophysiological characteristics of FSI, which is parvalbumin-positive. Scale bar, 50 µm. **B & E.** Sample traces depicting APs elicited in response to the depolarizing current injection in a CA2 PC (**B**) and a CA2 FSI (**E**). Scale bars: 20 mV/100 ms. **C & F**. Summary graphs with input-output curves of action potentials generation in vehicle- and ChABC-treated CA2 PCs (**C**; Vehicle: n=11, ChABC: n=6) and CA2 FSIs (**F**; Vehicle: n=7, ChABC: n=6). **D & G**. Depolarization thresholds of action potential in CA2 PCs (**D**) and CA2 FSIs (**G**). P>0.05, t-test, compared with vehicle. No effect of ChABC was detected.

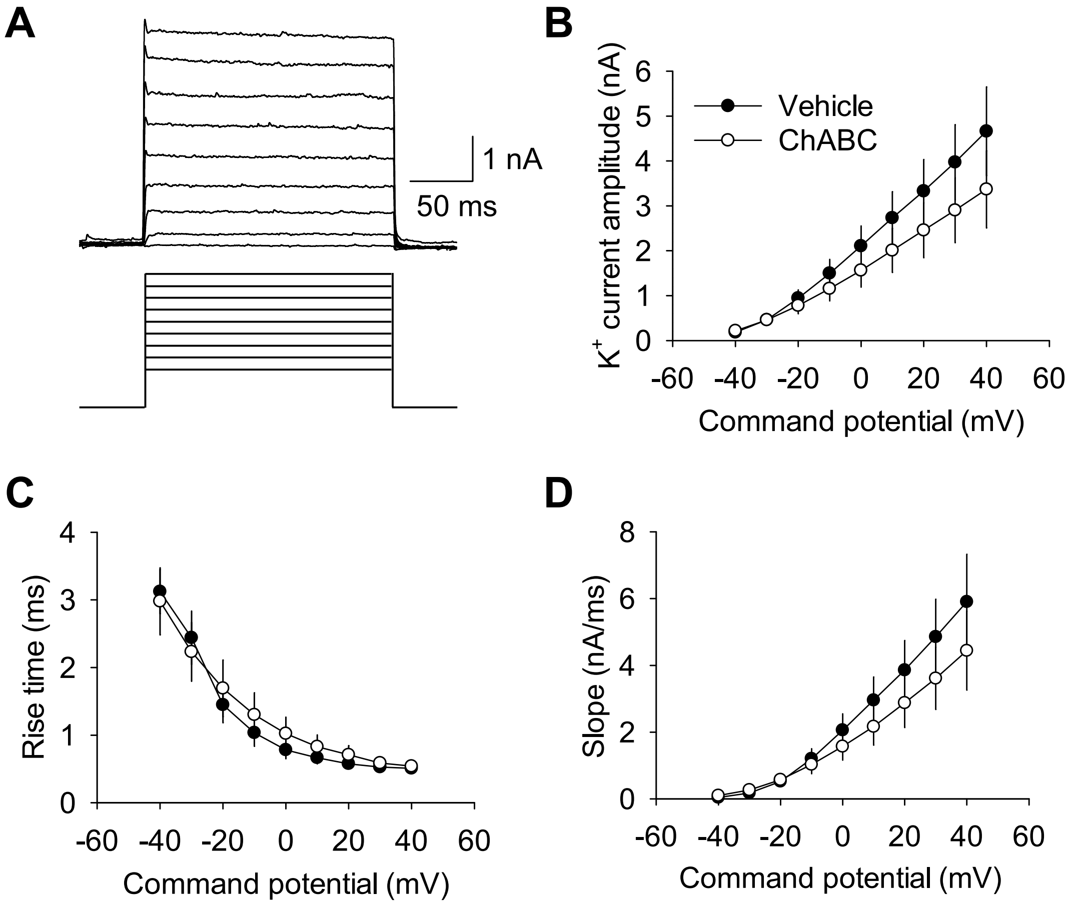

Supplemental Figure S2. Whole-cell potassium (K^+) current is not altered in CA2 fast-spiking interneurons seven days after injection of ChABC. A. Sample traces (from vehicle treated FSI) of outward potassium currents recorded in the CA2 FSIs in response to multiple voltage steps. **B-D**. Summary graphs for the peak amplitude of potassium currents (B), rise time of potassium currents (**C**), rising slope of potassium currents (**D**). Shown are means ± SEMs of the measured values. Vehicle: n=10, ChABC: n=10. No difference ($P > 0.05$) was detected between vehicle and ChABC-treated groups, repeated measures two-way ANOVA.

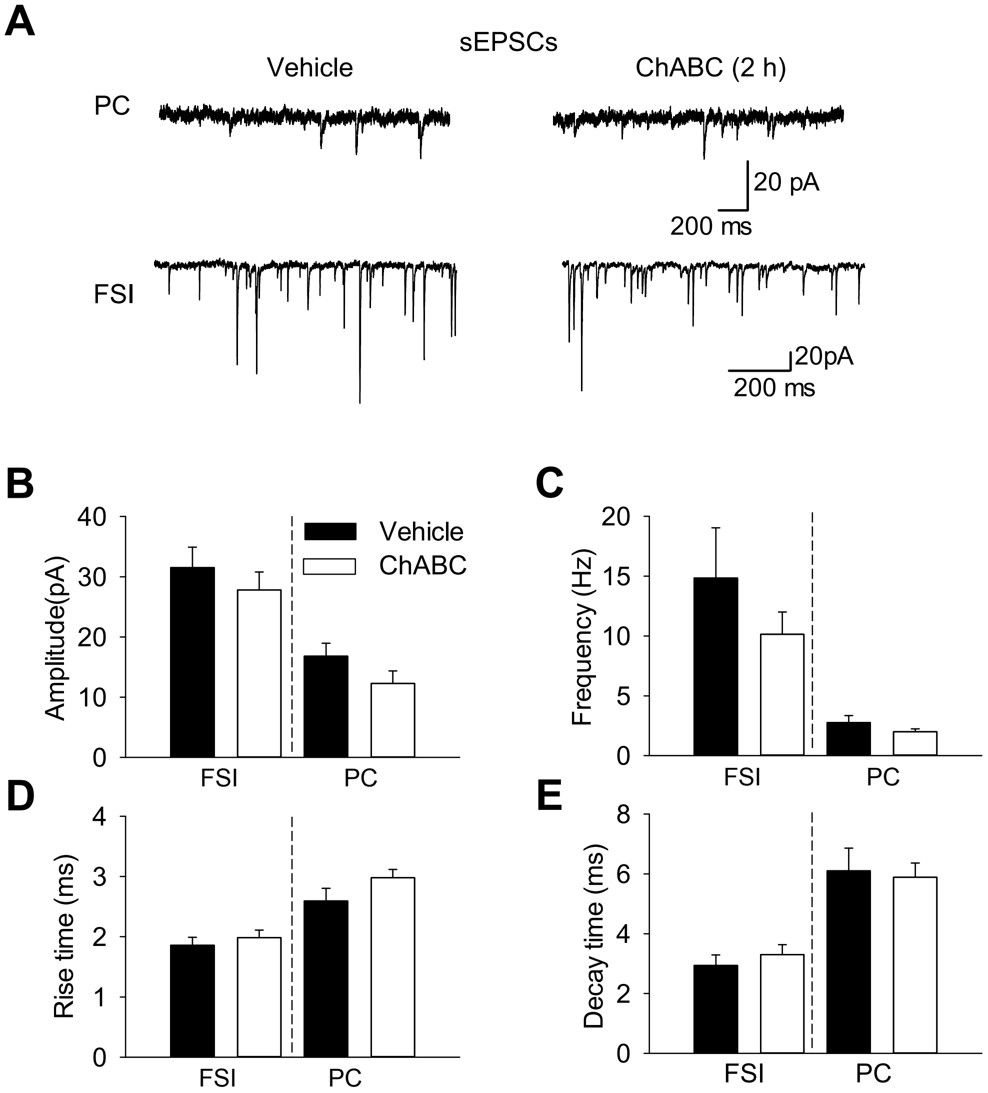

Supplemental Figure S3. Spontaneous excitatory synaptic transmission (sEPSCs) onto CA2 neurons is not altered after 2-hour ChABC treatment. **A**. Representative traces of sEPSCs recorded from CA2 PCs and FSIs in vehicle- and ChABC-treated hippocampal slices. **B**. Mean amplitude of sEPSCs. **C**. Frequency of sEPSCs. **D**. Rise time of sEPSCs. **E**. Decay time of sEPSCs. PCs: vehicle n=6, ChABC n=8; FSIs: vehicle n=9, ChABC n=10; $P > 0.05$ compared to vehicle, two-tailed t-test.

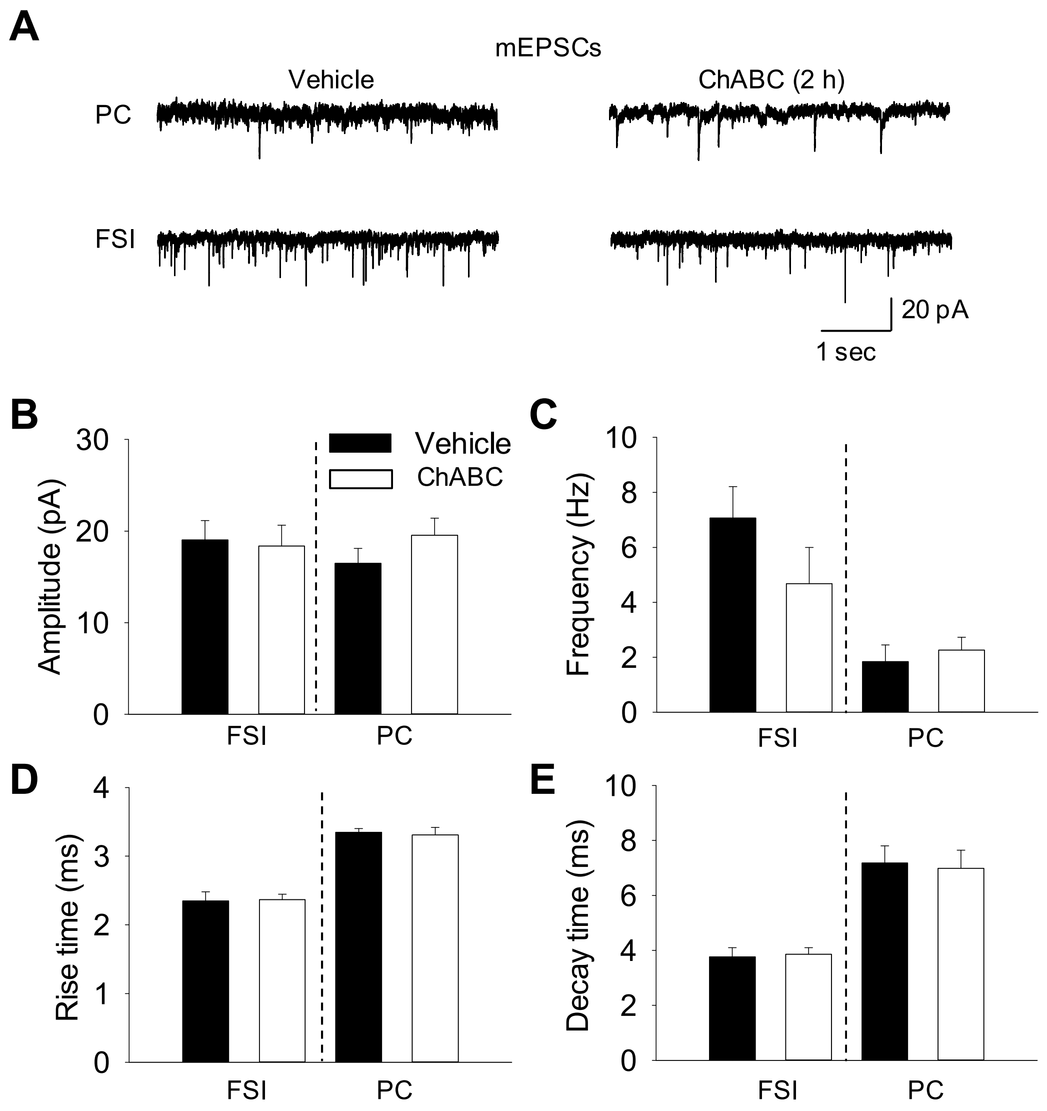

Supplemental Figure S4. Properties of mEPSCs after 2 hr ChABC treatment in CA2 neurons. Representative traces (**A**), amplitude (**B**), frequency (**C**), rise (**D**) and decay time (**E**) of mEPSCs in two different cells types after acute treatment of vehicle (PC: n=12; FSI: n=13) or ChABC (PC: n=8; FSI: n=10). Bars represent means ±SEMs in vehicle- (black) and ChABC-injected (white) mice. P > 0.05, unpaired t-test compared with vehicle.

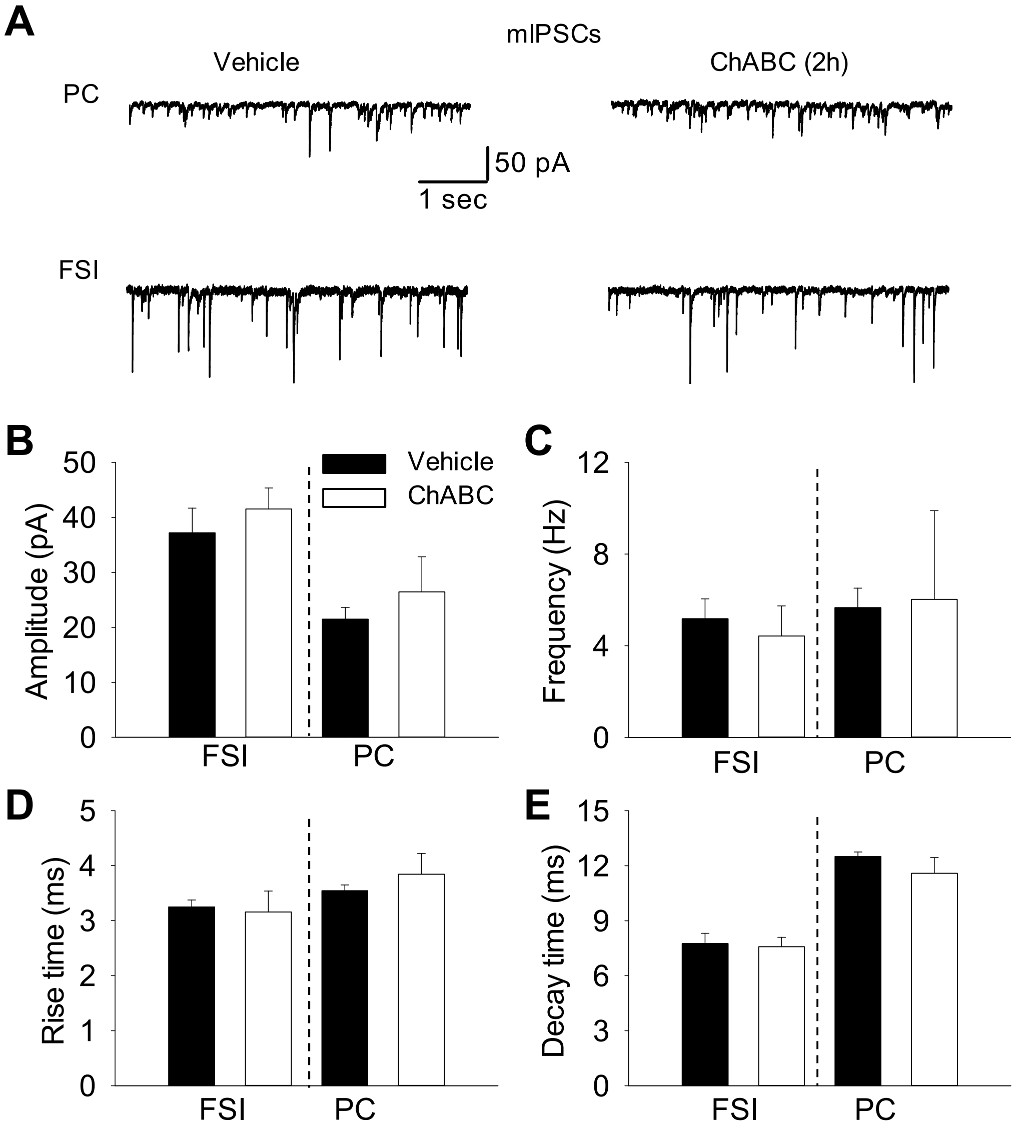

Supplemental Figure S5. Action potential-independent GABAergic transmission (mIPSCs) onto CA2 neurons after acute enzymatic removal of PNNs. Representative traces (**A**), amplitude (**B**), frequency (**C**), rise (**D**) and decay time (**E**) of mIPSCs in two different cells types after acute treatment with vehicle (PC: n=11; FSI: n=10) or ChABC (PC: n=5; FSI: n=6). P > 0.05, two-tailed t-test compared with vehicle.
